# Structural Basis for Inhibiting Porcine Epidemic Diarrhea Virus Replication with the 3C-Like Protease Inhibitor GC376

**DOI:** 10.3390/v12020240

**Published:** 2020-02-21

**Authors:** Gang Ye, Xiaowei Wang, Xiaohan Tong, Yuejun Shi, Zhen F. Fu, Guiqing Peng

**Affiliations:** 1 State Key Laboratory of Agricultural Microbiology, College of Veterinary Medicine, Huazhong Agricultural University, Wuhan 430070, China; yegang@webmail.hzau.edu.cn (G.Y.); wxw9535@webmail.hzau.edu.cn (X.W.); xiaohantong@whu.edu.cn (X.T.); shiyuejun2017@mail.hzau.edu.cn (Y.S.); zhenfu@uga.edu (Z.F.F.); 2 Key Laboratory of Preventive Veterinary Medicine in Hubei Province, The Cooperative Innovation Center for Sustainable Pig Production, Wuhan 430070, China; 3 Department of Pathology, College of Veterinary Medicine, University of Georgia, Athens, GA 30602, USA

**Keywords:** coronavirus, PEDV 3CL^pro^, inhibitor, crystal structure, complex

## Abstract

Porcine epidemic diarrhea virus (PEDV), being highly virulent and contagious in piglets, has caused significant damage to the pork industries of many countries worldwide. There are no commercial drugs targeting coronaviruses (CoVs), and few studies on anti-PEDV inhibitors. The coronavirus 3C-like protease (3CL^pro^) has a conserved structure and catalytic mechanism and plays a key role during viral polyprotein processing, thus serving as an appealing antiviral drug target. Here, we report the anti-PEDV effect of the broad-spectrum inhibitor GC376 (targeting 3Cpro or 3CL^pro^ of viruses in the picornavirus-like supercluster). GC376 was highly effective against the PEDV 3CL^pro^ and exerted similar inhibitory effects on two PEDV strains. Furthermore, the structure of the PEDV 3CL^pro^ in complex with GC376 was determined at 1.65 Å. We elucidated structural details and analyzed the differences between GC376 binding with the PEDV 3CL^pro^ and GC376 binding with the transmissible gastroenteritis virus (TGEV) 3CL^pro^. Finally, we explored the substrate specificity of PEDV 3CL^pro^ at the P2 site and analyzed the effects of Leu group modification in GC376 on inhibiting PEDV infection. This study helps us to understand better the PEDV 3CL^pro^ substrate specificity, providing information on the optimization of GC376 for development as an antiviral therapeutic against coronaviruses.

## 1. Introduction

Porcine epidemic diarrhea virus (PEDV), which was first observed in Europe in 1971 [1], belongs to the genus *Alphacoronavirus* in the family *Coronaviridae* and causes severe diarrhea, vomiting, dehydration and high mortality in neonatal piglets, resulting in severe agricultural loss [2,3]. Since PEDV was first identified, outbreaks have been reported in many swine-producing countries, notably in Europe and Asia. Before 2010, several commercial vaccines were widely used to control the spread of PEDV in Asia [2]. However, since 2010, frequent outbreaks of highly virulent strains of PEDV have occurred in Asia, and these outbreaks have been particularly severe in China [4,5,6]. The vaccines based on classic strains failed to control infection [2]. Furthermore, a vaccine-resistant PEDV strain emerged in the United States for the first time and spread rapidly throughout the country, causing large economic losses in the swine industry [3]. Subsequently, North America, Europe and Asia have also been attacked by a new PEDV strain [2,7]. Although newly developed vaccines provide efficient protection, vaccine resistance may still appear in the future due to the genetic diversity of epitopes among different virus genotypes [8]. Thus, it is necessary to develop other strategies to protect against PEDV infection.

Like all the other coronaviruses (CoVs), PEDV has a RNA genome that encodes two polyproteins, pp1a and pp1ab [9,10,11]. The majority of synthesized polyproteins are cleaved by 3C-like protease (3CL^pro^; main protease, Nsp5) at 11 conserved sites, which are essential for viral replication [10,12,13]. Thus, 3CL^pro^ is an appealing target in the design of anticoronavirus therapies [14,15,16,17,18]. Notably, the 3CL^pro^ proteins of different coronaviruses share higher sequence identity and have a more conserved catalytic core than the spike proteins of the same coronaviruses [19,20,21,22], which makes 3CL^pro^ an ideal broad-spectrum antiviral target [23,24,25,26]. Furthermore, two highly pathogenic human coronaviruses, severe acute respiratory syndrome coronavirus (SARS-CoV) and Middle East respiratory syndrome coronavirus (MERS-CoV), that both came from animals made people aware of the importance of the cross-species transmission of coronaviruses [27,28,29,30]. Since people have easy access to swine, the potential for cross-species transmission of PEDV should not be ignored. Therefore, it is necessary to develop new anti-PEDV therapies.

The coronavirus 3CL^pro^ employs conserved cysteine and histidine residues, which serve as the principal nucleophile and general acid–base catalyst, respectively, at its catalytic site [9,10,31]. 3CL^pro^ recognizes a conserved cleavage site containing a hydrophobic residue (preferably L) at the P2 position, a Q at the P1 position, and a small aliphatic amino acid residue (S, G or A) at the P1′ position [10,32,33]. Research breakthroughs in 3CL^pro^ structures have made it possible to design specific inhibitors against coronavirus replication, and some of these inhibitors show excellent broad-spectrum anti-coronavirus effects [17,23,25,26,31,34,35]. GC376, a dipeptidyl bisulfite adduct salt, exerts an excellent inhibitory effect on some picornaviruses and coronaviruses in enzymatic or cell-based assays [35,36,37]. Notably, antiviral treatment with GC376 leads to a full recovery in laboratory cats with feline infectious peritonitis (FIP), a highly fatal feline disease caused by feline coronavirus (FIPV) that has no commercial vaccines or drugs [18]. In addition, PEDV 3CL^pro^ shares a 62% sequence identity with FIPV 3CL^pro^.

In this study, we detected the inhibitory effects of GC376 on the PEDV 3CL^pro^. The antiviral effects of the inhibitor were studied using two PEDV strains, namely, CV777 and YN144. Finally, we determined the structure of the PEDV 3CL^pro^ in complex with GC376 and compared this structure with other available 3CL^pro^ structures. Our study provides a structural model for the development of anti-PEDV drugs and further enriches structural knowledge for the optimization of broad-spectrum anticoronavirus drugs.

## 2. Materials and Methods

### 2.1. Compound

**Figure 1:**
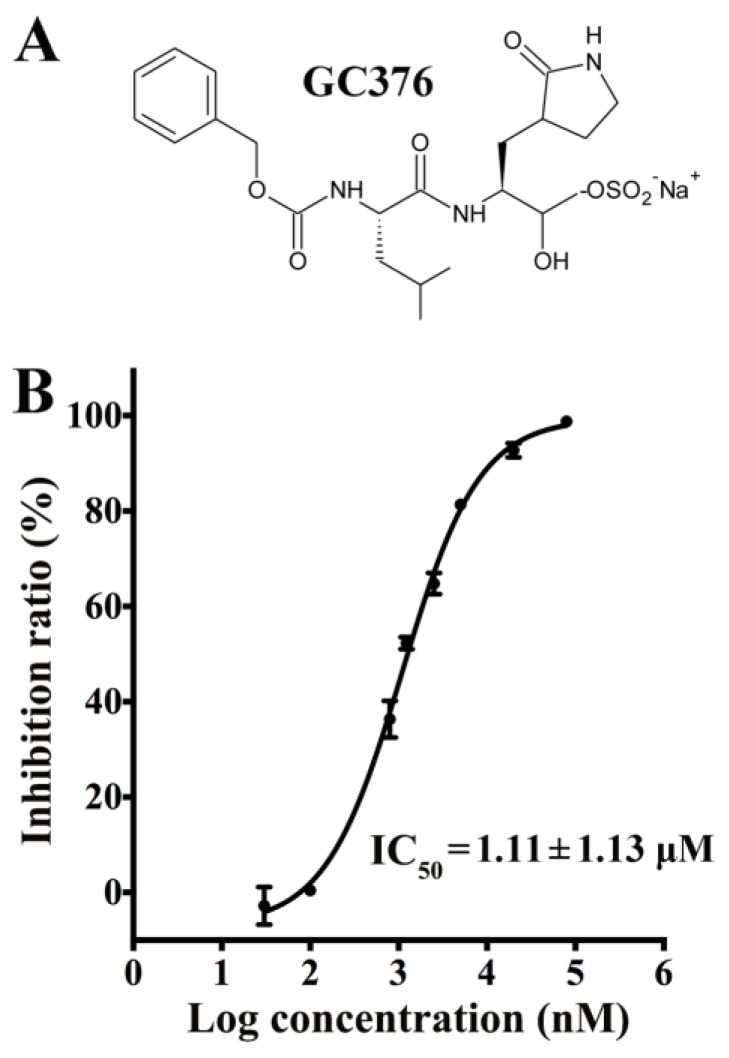
Structure of GC376 and the inhibitory effect of GC376 on the porcine epidemic diarrhea virus (PEDV) 3CL^pro^ determined by FRET assays. (**A**) GC376 is a dipeptidyl bisulfite adduct salt that showed excellent inhibitory effects on some picornaviruses and coronaviruses in enzymatic or cell-based assays and (**B**) an enzyme (100 nM) and a fluorescent peptide substrate (40 μM) were used in the FRET assay, and the concentration of GC376 ranged from 0.03 μM to 80 μM. The 50% inhibitory concentration (IC_50_) was calculated using GraphPad Prism5 to assess inhibition ratios at different inhibitor concentrations. The error bars show the S.D. of the results from three replicates.

GC376 (Figure 1A) and its two variants Target 1 and Target 2 were synthesized at LabNetwork (global e-Commerce platform for discovery compound and building block: www.labnetwork.com, Shanghai 200131, China) with purity higher than 95%. In Target 1, Leu of GC376 is replaced with Met, which has a longer side chain; in Target 2, Leu of GC376 is replaced with Phe, which has a side chain with a larger volume. The compound was dissolved in dimethyl sulfoxide (DMSO) at a concentration of 100 mM and stored at −80 °C.

### 2.2. Viruses and Cells

African green monkey cells (Vero cells; Cat. No. CCL81, ATCC) were cultured with Dulbecco’s minimum essential medium (HyClone, Pittsburgh, PA, USA) supplemented with 10% fetal bovine serum (Gibco, Waltham, MA, USA). The viruses used in this study were the attenuated strain YN144 (*Accession No. KT021232*, [38]) and the prototype strain CV777 [39]. Notably, 5 μg/mL trypsin was added to the medium during culture of the YN144 virus.

### 2.3. Expression and Purification of the PEDV 3CL^pro^

The strategy for plasmid construction, protein expression and purification of the PEDV 3CL^pro^ was described in a previous study [40]. Briefly, the coding sequence (from the FJZZ strain) with a C-terminal His_6_-tag was inserted into the pET42b (+) vector. Proteins were induced with 1 mM isopropyl-β-d-thiogalactopyranoside (IPTG) at 27 °C for 7 h. A His TrapTM HP column (GE Healthcare, Pittsburgh, PA, USA) and a 120-mL Superdex 200 (GE Healthcare, Pittsburgh, PA, USA) column were used for protein purification.

### 2.4. Förster Resonance Energy Transfer (FRET)-Based Assays for 50% Inhibitory Concentration (IC_50_) Measurement

The fluorescent peptide substrate “Dabcyl-YNSTLQ↓AGLRKM-E-Edans” (GenScript, Piscataway, NJ, USA), which contains the N-terminal cleavage site of the PEDV 3CL^pro^, was used in FRET assays. All reactions were performed in a solution of 20 mM HEPES, 50 mM NaCl, 0.4 mM EDTA, 30% glycerol and 4 mM DTT at pH 8.0 in a total volume of 100 μL. The enzyme concentration used in FRET assays was 100 nM, and the substrate concentration was 40 μM. The concentrations of GC376 ranged from 0.03 μM to 80 μM. The fluorescence units upon cleavage were monitored every 5 min for 30 min at 485 nm, with excitation at 340 nm. Relative fluorescence units (RFU) were determined by subtracting the background value (substrate control well without the protease) from the fluorescence values. Finally, the 50% inhibitory concentration (IC_50_) was calculated using GraphPad Prism5 (San Diego, CA, USA) to assess the inhibition ratios at different inhibitor concentrations.

### 2.5. Cell-Based Assays for Concentration for the 50% Maximal Effect (EC_50_) Measurement

Before the 50% maximal effect (EC_50_) measurement, the 50% cell death toxic concentrations (CC_50_) of GC376 in Vero cells were determined. Briefly, confluent cells grown in 96-well plates were treated with various concentrations (50–200 μM) of GC376 for 72 h. All the wells, including control wells, contained 0.2% DMSO. Cell cytotoxicity was measured using the Celltiter-Glo Luminescent Cell Viability Assay reagent (Promega, Madison, WI, USA). For EC_50_ measurement, two PEDV strains (CV777 and YN144) were used. When cells were grown to 80%–90% confluence, the virus was added at a multiplicity of infection (MOI) of 0.01 with a series of GC376 concentrations (0.78 μM, 1.56 μM, 3.13 μM, 6.25 μM, 12.5 μM, 25 μM, 50 μM and 100 μM). All the wells, including the virus and cell control wells, contained 1% DMSO. Cell viability was measured as above at 12 h and 36 h for YN144 and CV777, respectively. Finally, the EC_50_ was calculated using GraphPad Prism5 (San Diego, CA, USA) to assess the inhibition ratios at different inhibitor concentrations.

### 2.6. Western Blot Analysis

Vero cells were seeded in 6-well plates at a density of 1 × 10^6^ cells per well. When cells had grown to 80%–90% confluence, the virus was inoculated into the cells at an MOI of 0.01 with the same GC376 concentrations as those used for the EC_50_ measurements. After incubating at 37 °C for 12 h and 36 h for YN144 and CV777 respectively, the cells were lysed in a lysis buffer containing protease inhibitors (Beyotime, Shanghai, China) on ice. The supernatants were used for Western blot assays. After SDS-PAGE, the proteins were transferred to PVDF membranes (Bio-Rad, Hercules, CA, USA). After blocking with 5% skimmed milk at room temperature for 3 h, the membranes were incubated with an anti-PEDV nucleocapsid (N) protein monoclonal antibody and an anti-GAPDH monoclonal antibody (Proteintech, Rosemont, IL, USA) for 2 h. Then, the membranes were incubated with an HRP-conjugated secondary antibody (Boster Biological Technology, Pleasanton, CA, USA) for 40 min. The membranes were visualized using an enhanced chemiluminescence system (Amersham Imager 600, GE Healthcare, Pittsburgh, PA, USA), and the nucleocapsid protein level was normalized to the GAPDH protein level.

#### 2.6.1. Indirect Immunofluorescence Assay

Vero cells were seeded in 96-well plates at a density of 1 × 10^4^ cells per well. YN144 was inoculated into the wells at an MOI of 0.01, and GC376 was added at a series of concentrations at the same time. Indirect immunofluorescence assays were performed when 60% of the cells died. Briefly, cells were washed with PBS before fixation by 4% formaldehyde in PBS, treated with 0.1% Triton X-100, and blocked with 2% BSA. After blocking, the cells were incubated with the primary antibody and a FITC-labeled secondary antibody (ThermoFisher, Waltham, MA, USA) in the dark. Finally, the cells were examined under fluorescence microscopy after staining with DAPI.

#### 2.6.2. Crystallization and Structure Determination

The crystal of the PEDV 3CL^pro^ for GC 376 soaking was obtained as described in a previous study [40]. GC376 was dissolved in elution buffer B (20 mM Tris, pH 7.4, 200 mM NaCl) at a concentration of 6 mM and then added to the crystallization drop at an equal volume. The crystals were soaked for 16 h before being flash-frozen. The single crystals were first washed with 5%, 10%, 15% and 30% ethylene glycol (*v*/*v*) as a cryoprotectant and then flash-frozen in liquid nitrogen. All data collection was performed at beamline BL17U at the Shanghai Synchrotron Radiation Facility (SSRF) using a MAR 225 CCD detector (MAR Research). The data sets were indexed, integrated and scaled using HKL-3000 (Charlottesville, VA, USA) [41]. The structure was solved by molecular replacement with PHASER (Phenix, Berkeley, CA, USA) [42] using the structure of the PEDV 3CL^pro^ (PDB identifier 4XFQ) as a starting model. Manual model building was performed using Coot (Oxon, UK) [43], and the structure was refined with Phenix (Berkeley, CA, USA) [44]. Refinement statistics are shown in Table 1. All of the structural figures were drawn using PyMOL (Schrödinger). Coordinate and structure factors have been submitted to the PDB (accession number 6L70).

## 3. Results

### 3.1. Inhibitory Effects of GC376 on the PEDV 3CL^pro^

Compared with the sequence of 3CL^pro^ from the FJZZ strain, the sequences for 3CL^pro^ from the YN144 strain and CV777 strain were identified to differ by one and six residues, respectively. All of the different residues were far from the substrate-binding site of the PEDV 3CL^pro^ (PDB: 4ZUH, FJZZ strain) and might not cause obvious differences in enzymatic activities; thus, we used the PEDV FJZZ 3CL^pro^ stored in our laboratory for inhibition assays. The results indicated that GC376 exerted strong inhibitory effects on the PEDV 3CL^pro^ with an IC_50_ of approximately 1.11 μM (Figure 1), which was comparable to the IC_50_ values of other coronaviruses reported in a previous study [35].

### 3.2. Antiviral Effects of GC376 on the PEDV CV777 and YN144 Strains

**Figure 2:**
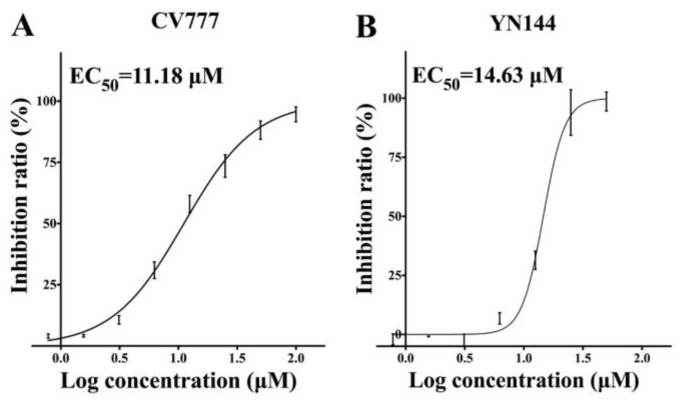
Antiviral effects of GC376 on the replication of two PEDV strains in cell culture. (**A**) CV777 was added at a multiplicity of infection (MOI) of 0.01 with a series of GC376 concentrations (0.78 μM, 1.56 μM, 3.13 μM, 6.25 μM, 12.5 μM, 25 μM, 50 μM and 100 μM). Cell viability was measured using Celltiter-Glo Luminescent Cell Viability Assay reagent (Promega, Madison, WI, USA) at 36 h. Finally, the 50% maximal effect (EC_50_) values were calculated using GraphPad Prism 5 to assess inhibition ratios at different inhibitor concentrations. The error bars show the S.D. of the results from three replicates. (**B**) The EC_50_ of YN144 was measured as above except that the cell viability was measured at 12h. The error bars show the S.D. of the results from three replicates.

Before determining the inhibitory effects of GC376 on viruses in Vero cells, we tested the cytotoxicity of the compound. GC376 had no cytotoxicity up to a concentration of 200 μM (data not shown). GC376 at a series of concentrations was used to evaluate the suppression of two PEDV strains. The EC_50_ values of GC376 against CV777 and YN144 were similar, namely, 11.18 μM and 14.63 μM, respectively (Figure 2). The results imply that GC376 should efficiently inhibit the replication of different PEDV strains, possibly because of the conserved catalytic core of 3CL^pro^. It is also notable that YN144 caused a cytopathic effect (CPE) much faster than CV777 (12 h and 36 h respectively); however, GC376 showed similar inhibitory effects with different treatment times for the two viruses, which indicates that GC376 quickly enters the cell and blocks viral 3CL^pro^.

**Figure 3:**
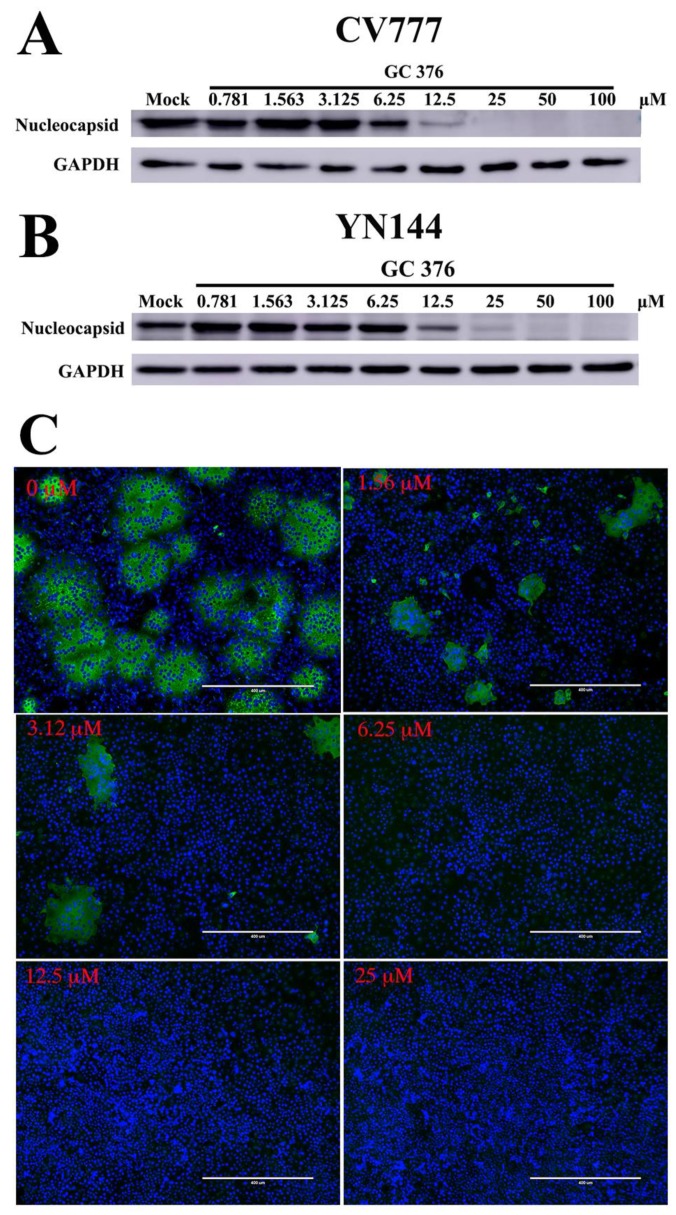
Western blot analysis of the effects of GC376 on the replication of two PEDV strains in cell culture. (**A**,**B**) Vero cells were infected with one of two PEDV strains at an MOI of 0.01 and then treated with GC376 at different concentrations. Cell extracts were analyzed by western blotting for nucleocapsid expression, and GAPDH was used as an internal control. (**C**) Indirect immunofluorescence assays are shown. The white bar measure is 400 μm. YN144 was used to infect cells at an MOI of 0.01, and GC376 was added to the cells at a series of concentrations. The cells were treated with primary antibody (anti PEDV N) and then a FITC-labeled secondary antibody. The cells infected by viruses were captured in green as shown in the picture.

Western blot assays were introduced to further quantify the antiviral effects of GC376 on the two viruses. Both of the viral nucleocapsid proteins were decreased in a similar dose-dependent manner with increasing GC376 concentrations (Figure 3A,B). The N proteins showed significant reductions at 12.5 μM and were almost undetectable at 25 μM. The results further indicated that GC376 had similar inhibitory effects on the two PEDV virus strains with an IC_50_ of approximately 12.5 μM, which was consistent with that of the cell-based IC_50_ measurement assays. Since GC376 showed similar effects against the two PEDV strains, we performed IFA with one of the PEDV strains, YN144. Compared with the non-drug-treated cells, GC376 reduced virus infection at 1.56 μM (Figure 3C). It almost completely inhibited virus replication at approximately 6.25 μM, and cell syncytosis disappeared; 12.5 μM GC376 abolished the infection.

### 3.3. GC376 Inhibits PEDV Replication by Blocking the Catalytic Residues and Binding Pocket of 3CL^pro^

**Figure 4:**
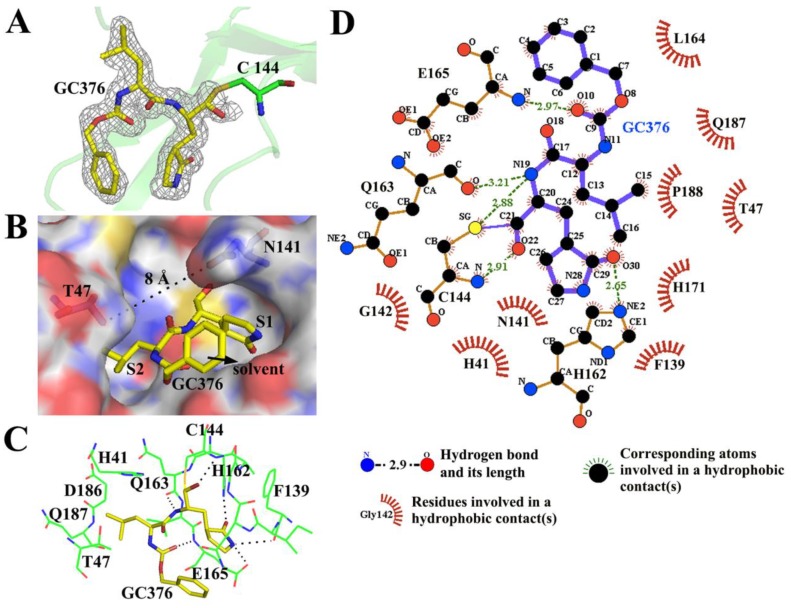
Crystal structure of the PEDV 3CL^pro^ in complex with GC376. (**A**) Electron density map of GC376 (2Fo-Fc, contoured at 1.0 σ). The bisulfite group of GC376 was removed, and the compound formed a covalent bond with Cys144; (**B**) three-dimensional structure of the substrate-binding pockets occupied by GC376. The pocket is shown as the surface, and the two residues Thr 47 and Asn 141 are shown as sticks. The distance between the two residues is labeled. GC376 is shown in yellow as sticks. The glutamine surrogate ring and the leucine of GC376 fit comfortably into the S1 and S2 binding sites, respectively. The 6-membered aromatic ring of GC376 points to the solvent; (**C**) diagram of the detailed molecular interactions between GC376 and the protease. The residues of the pockets are represented in green as lines, and GC376 is shown in yellow as sticks. Hydrogen bond interactions are shown as black dashed lines; (**D**) structural diagram of the distributions of hydrophobic and hydrophilic interactions at the interface of the PEDV 3CL^pro^ in complex with GC376. The residues of the protease are shown as orange sticks and red arcs with spokes. GC376 is shown in blue as sticks. Carbon, nitrogen, oxygen and sulfur atoms are shown as black, blue, red and yellow circles, respectively. Hydrogen bonds are shown as green dashed lines labeled with the distance between the donor atom and corresponding acceptor atom. Hydrophobic interactions are demonstrated by arcs with spokes radiating toward the atoms (with spokes around) or residues (shown as arcs with spokes) they contact.

To elucidate the inhibitory mechanism and provide a structural model for further inhibitor optimization of GC376 against the PEDV 3CL^pro^, we determined the structure of the 3CL^pro^–GC376 complex (1.65 Å). Investigation into the substrate-binding site revealed a prominent electron density for GC376 in two monomers, especially in subunit A (Figure 4A). The interface area between the PEDV 3CL^pro^ and GC376 was 373 Å^2^, and the buried surface of GC376 took up 76% of the total solvent-accessible area (computed using the PDBePISA tool, http://pdbe.org/pisa/). As the 6-membered aromatic ring of GC376 occupied the place of the PEDV 3CL^pro^ substrate P3 residue, whose side chain usually points to the solvent, it contributed little to the interface (Figure 4B). The glutamine surrogate ring and the leucine of GC376 fit comfortably into the S1 and S2 binding sites, respectively. However, in subunit B, there was no density for the 6-membered aromatic ring of GC376, and only part of the density was available for the glutamine surrogate ring (data not shown). These detailed interactions were analyzed using PyMOL and the LigPlot^+^ program (Figure 4). The bisulfite group of GC376 was removed, and the compound formed a covalent bond with Cys 144. Hydrophobic interactions were extensive between the protease and the compound (Figure 4D). Four residues of the PEDV 3CL^pro^, Cys 144, His 162, Gln 163 and Glu 165, are involved in the hydrogen bond interactions with GC376 (Figure 4C,D).

Superposition of the PEDV 3CL^pro^-GC376 complex with the previously reported apo-PEDV 3CL^pro^ structure (PDB: 4XFQ) yielded root mean square deviations (RMSDs) of 0.31 Å and 0.18 Å between the Cα atoms for chains A and B, respectively. This finding indicated that no large conformational change in the overall structure occurred upon ligand binding. However, examination of the substrate-binding pocket between chains A and B of the PEDV 3CL^pro^-GC376 complex revealed a structural difference in the loop composed of residues 44–52 (ASSTTSTID). Interestingly, there was only electron density (contoured at 1.0 σ) for the 46–48 residues (STT) of chain A but not for chain B. This result suggests that the part of the loop is flexible and that its orientation directly influences the entrance size of the substrate-binding pocket. Thr 47 in the loop and Asn 141, which is located on another side of the binding pocket, both protrude into the pocket, forming a narrow entrance with the width of ~8 Å (Figure 4B). However, the size of the entrance may change due to the flexibility of the loop during substrate cleavage.

### 3.4. Substrate Specificity of PEDV 3CL^pro^ at the P2 Site and the Optimization of GC376

**Figure 5:**
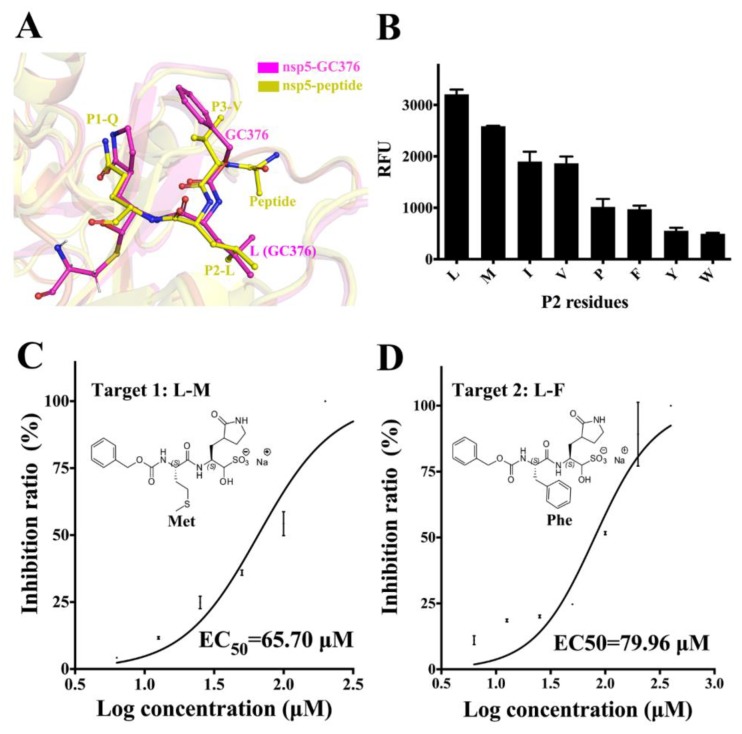
Substrate specificity of the PEDV 3CL^pro^ at the P2 site and the optimization of GC376. (**A**) The structure of PEDV 3CL^pro^-GC376 (light magenta) is superimposed over the complex structure of PEDV 3CL^pro^-peptide (yellow, PDB: 4ZUH). GC376 and the peptide substrate are shown as sticks with balls. The P1, P2 and P3 residues of the substrate and the Leu residue of GC376 are shown and labeled; (**B**) an enzyme (100 nM) and fluorescent peptide substrates (40 μM) were used in the FRET assay. The relative fluorescence units (RFU) detected upon cleavage of the fluorescent peptide substrate and its seven variants by the PEDV 3CL^pro^ are shown in the figure; (**C**,**D**) viability of YN144-infected Vero cells after treatment with the two GC376 variants, Target 1 (L-M) and Target 2 (L-F), was measured using the Celltiter-Glo Luminescent Cell Viability Assay reagent (Promega, Madison, WI, USA). EC_50_ values were calculated using GraphPad Prism5 to assess inhibition ratios at different inhibitor concentrations. The structures of the two compounds are shown in the figure, and their modification sites are labeled. All the error bars show the S.D. of the results from three replicates.

Superimposition of the PEDV nsp5-GC376 and PEDV nsp5-peptide complex (PDB: 4ZUH) [40] indicated that the glutamine surrogate ring and the leucine of GC376 perfectly occupy the position of the P1-Q and P2-L residues of the peptide substrate, respectively (Figure 5A). As P1-Q is the most conserved residue of the PEDV 3CL^pro^ recognition sequence, there may be few possibilities to introduce modifications at this site, or doing so may be difficult. In examining all the P2 residues of the cleavage site in the PP1ab, two different residues were observed (Val and Met). To determine the preferred P2 residues of PEDV 3CL^pro^, fluorescent peptide substrates with different P2 residues were synthesized. Except for Val and Met, several other residues with different side chain sizes were introduced into the substrates (Figure 5B). FRET assays using the different substrates were performed as described above. The original substrate without P2 mutation exhibited the highest cleavage efficiency. P2-M showed a little decrease, and P2-I and P2-V retained 60% activity. Furthermore, a large reduction of approximately 70% compared to that of P2-L was observed for P2-P/Y/ F/W. Thus, Leu is the most preferred residue at P2 site of PEDV 3CL^pro^.

To explore whether replacement of the P2-Leu of GC376 with different residues could improve the inhibitory effects, two GC376 variants were synthesized. In Target 1, Leu is replaced with Met, which has a longer side chain; in Target 2, Leu is replaced with Phe, which has a side chain with a larger volume. The EC_50_ of the two compounds fir YN144 infection was measured to evaluate optimization effects. Targets 1 and 2 yielded EC_50_ values of 65.7 μM and 80.0 μM respectively (Figure 5C,D). Both showed higher EC_50_ values compared to GC376, which has a leucine. As a consequence, GC376 with Leu shows the highest inhibitory effects on PEDV infection.

### 3.5. Comparison between the PEDV 3CL^pro^–GC376 Complex and TGEV 3CL^pro^–GC376 Complex

**Figure 6:**
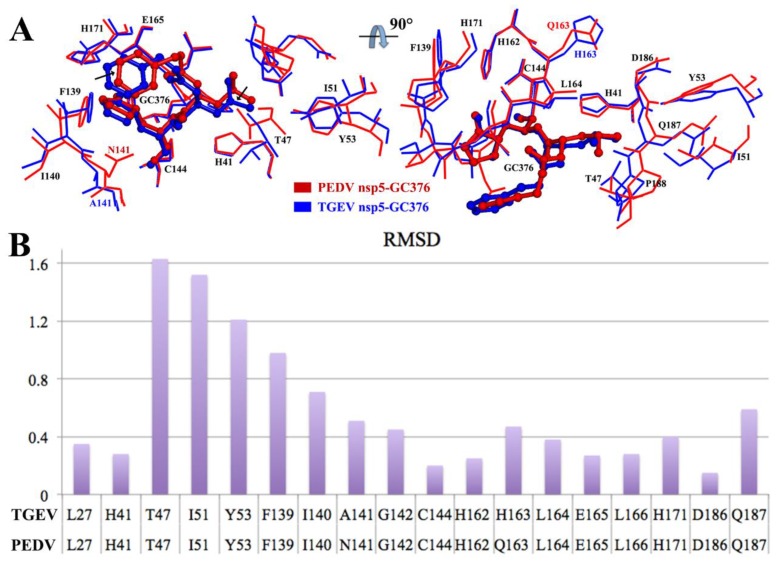
Structure alignment between the PEDV 3CL^pro^–GC376 and transmissible gastroenteritis virus (TGEV) 3CL^pro^–GC376. (**A**) The structure of PEDV 3CL^pro^–GC376 (red) is superimposed with the complex structure of TGEV 3CL^pro^–GC376 (blue). GC376 is shown as sticks with balls, and the residues of 3CL^pro^ involved in binding with GC376 are labeled and shown as lines. The conserved residues between the two 3CL^pro^s are labeled in black and nonconserved residues are labeled in different colors. The black arrows indicate the structural deviation of the Leu side chain and 6-membered aromatic ring of GC376 between the two structures and (**B**) the root mean square deviation (RMSD) values for the residues involved in the GC376 binding of the two structures.

It is reported that GC376 shows excellent antiviral activities against the transmissible gastroenteritis virus (TGEV), and the structure of TGEV 3CL^pro^–GC376 complex is determined [35]. We compared the binding between the PEDV 3CL^pro^–GC376 complex and TGEV-3CL^pro^–GC376 complex. Most of the residues forming the substrate-binding pocket were conserved, and the two 3CL^pro^s showed a highly similar binding mode for GC376 (Figure 6A). The RMSD values of the most listed residues between the two proteases were approximately 0.4 Å, and three residues from a loop (T47, I51 and Y53) showed RMSD values higher than 1 Å (Figure 6B). Some of the nonconserved residues, such as Q163 of the PEDV 3CL^pro^ and H163 of the TGEV 3CL^pro^, formed similar hydrogen bonding interactions between the main chain oxygen and GC376. Overall, there were two relatively obvious differences: a. the loop (46-49) was stable, and water-mediated contacts were observed between Thr 47 and GC376 in the TGEV 3CL^pro^–GC376 complex, which have also been found in the norovirus (NV) 3CL^pro^ and picornavirus (PV) 3CL^pro^ in complex with GC376 [35]; however, the loop (46-49) was flexible, and only hydrophobic interactions were observed in the PEDV 3CL^pro^-GC376 complex. b. The Asn 141 residue, which has a side chain that protrudes into the pocket, resulted in a narrower entrance in the PEDV 3CL^pro^ than that in the TGEV 3CL^pro^, which has an Ala 141 residue instead (Figure 6). The two differences cause small structural deviations in the Leu side chain and the 6-membered aromatic ring of GC376 between the two structures, respectively. At the same time, the differences influenced the size of the entrance to the pocket, which might affect the efficiency of the entry and release of the 3CL^pro^ substrate.

## 4. Discussion

Viral proteases play a vital role in the viral lifecycle and are confirmed to be ideal antiviral targets. Several remarkably potent drugs targeting individual viral proteases have been developed against important human pathogens, such as human immunodeficiency virus (HIV) and hepatitis C virus (HCV) [45,46,47,48].

This strategy is also applicable to coronaviruses, which encode a 3CL^pro^ responsible for viral polyprotein cleavage. Porcine epidemic diarrhea virus (PEDV), an α coronavirus, is one of the major threats to the swine industry worldwide. The development of antiviral drugs targeting 3CL^pro^ is an important strategy to prevent PEDV epidemics. The global outbreak of SARS in 2003 accelerated study on anti-coronavirus inhibitor development, and many researchers have been committed to developing broad-spectrum antiviral inhibitors against coronavirus 3CL^pro^.

In our study, a series of available coronavirus inhibitors were applied in FRET-based assays to identify an effective inhibitor of PEDV. GC376, a dipeptidyl bisulfite adduct salt, worked best and was selected as the subject of subsequent experiments. Biochemical assays yielded an IC_50_ of 1.11 ± 0.13 μM for GC376 against the PEDV 3CL^pro^, which was slightly higher than that against the transmissible gastroenteritis virus (TGEV) 3CL^pro^ (0.82 ± 0.47 μM) and lower than that against the SARS-CoV 3CL^pro^ (4.35 ± 0.47 μM) [35].

Subsequently, we detected the anti-PEDV effect of GC376 on cells. Two PEDV strains were selected for evaluation of their antiviral effects. Sequence alignment of the 3CL^pro^s from the CV777 and YN144 strains revealed seven different residues. However, both EC_50_ and Western blot assays showed similar antiviral effects for GC376 on the two virus strains (Figure 2 and Figure 3). The seven residues that differ between the CV777 and YN144 strains were not involved in the binding pocket and thus had no obvious influence on the antiviral effect. GC376 exhibits potent antiviral activities with high nanomolar EC_50_ values against TGEV and FIPV [35,49]. However, we obtained low micromolar EC_50_ values against both PEDV strains. We analyzed the binding details of the PEDV 3CL^pro^-GC376 complex and another available 3CL^pro^-GC376 structure from another coronavirus (TGEV). However, most of the residues involved in GC376 binding are conserved. Only the loop (46-49) and entrance size show some differences (Figure 6). However, considering that the activities of these proteins at the biochemical level are not as different as those at the cellular level, the structural differences may not be the only reason for the difference in GC376 antiviral activities between PEDV and TGEV (or FIPV) observed in the cellular assays. The antiviral effect is more complicated at the cellular level and may be affected by the expression level of 3CL^pro^, other viral replication characteristics, or the pharmacokinetics of GC376 in different host cells.

In determining the P2 residue specificity of PEDV 3CL^pro^ and optimization of GC376, Leu showed best effects in both assays (Figure 5). In FRET assays using peptide substrates with different P2 residues, P2-M with a longer side chain and P2-I/V with shorter side chains showed lower cleavage efficiency (Figure 5B). This suggests that the length of Leu fits best in the S2 subsite. P2-P/F, which have a larger pyrrolidine or benzyl side chain group, resulted in a great increase in activity, which indicates that the residue with a side chain volume larger than that of Leu is not acceptable at the S2 subsite, further proving that P2-Y/W with even larger side chains are almost uncleavable (Figure 5B). Two modified compounds, Target 1 and Target 2, both showed lower inhibitory effects compared to that of GC376. GC376 (L-M) had stronger effects than GC376 (L-P) (Figure 5C,D), which was consistent with the results of FRET-based P2-site specificity assays. In conclusion, Leu of GC376 was the most preferred group in the S2 subsite, and we could rationalize the design of it based on Leu in the future. Perhaps replacement of one of the hydrogens of the Leu side chain by other atoms that causes minor changes might produce stronger binding, thus resulting in better inhibition. The 6-membered aromatic ring of GC376 stretches into the solvent and may not serve as a primary modification site.

Further animal experiments are required to evaluate its therapeutic effects on porcine epidemic diarrhea (PED) in piglets. In general, GC376 exerted an excellent inhibitory effect on different PEDV strains by targeting its 3CL^pro^, which has great potential, and could be modified to produce strong therapeutic effects on animals. Our study provides more information for the optimization of inhibitors.

## Figures and Tables

**Table 1:** Data collection and refinement statistics.

	**PEDV 3CL^pro^ in Complex with GC376**
**Data Collection**	
Space group	P 1 2_1_ 1
Cell parameter (a, b, c(Å))	56.86, 92.22, 58.30
α, β, γ	90.00°, 100.09°, 90.00°
Wavelength	0.97918
Resolution range (Å)	46.11–1.56
% Completeness	98.6 (99.0)
R_merge_ (last shell)	0.055 (0.060)
I/σ (last shell)	17.0 (2.5)
CC (1/2)	0.999 (0.864)
Redundancy (last shell)	6.9 (7.0)
**Refinement**	
Resolution (Å)	39.80–1.56
R_work_/R_free_	0.174/0.207
No. reflections	90,828
No of protein atoms	8998
No. of solvent atoms	1014
No. of ions/ligands	2
**r.m.s.d.**	
Bond length (Å)	0.011
Bond angle (Å)	1.10
Average B factor (Å^2^)	29.34
Protein	28.75
Water	35.08
Ligand	41.97
Ramachandran plot: core, allow,	97.81%, 2.02%,
disallow	0.17%

Highest resolution values are written in parenthesis. R_merge_ = Σ Σ |Ii − <I>|/Σ Σ Ii; where is Ii the intensity measurement of reflection h and <I> is the average intensity from multiple observations. R_work_ = Σ ||Fo| − |Fc||/Σ |Fo|; where Fo and Fc are the observed and calculated structure factors respectively. R_free_ is the equivalent to R_work_ but where 5% of the measured reflections have been excluded from refinement and set aside for cross-validation.
